# Food Purchase Behavior during The First Wave of COVID-19: The Case of Hungary

**DOI:** 10.3390/ijerph19020872

**Published:** 2022-01-13

**Authors:** Tamás Madarász, Enikő Kontor, Emese Antal, Gyula Kasza, Dávid Szakos, Zoltán Szakály

**Affiliations:** 1 Institute of Sports Economics and Management, Faculty of Economics and Business, University of Debrecen, Böszörményi Street 138, H-4032 Debrecen, Hungary; madarasz.tamas@econ.unideb.hu; 2 Institute of Marketing and Commerce, Faculty of Economics and Business, University of Debrecen, Böszörményi Street 138, H-4032 Debrecen, Hungary; szakaly.zoltan@econ.unideb.hu; 3 Hungarian Platform of Diet, Physical Activity and Health, Andrássy Street 61, H-1062 Budapest, Hungary; diet.emese.antal@gmail.com; 4 National Food Chain Safety Office, Kitaibel Pál Street 1, H-1024 Budapest, Hungary; kaszagy@nebih.gov.hu (G.K.); kszakosd@nebih.gov.hu (D.S.)

**Keywords:** food purchase behavior, COVID-19, Hungary, pandemic

## Abstract

Coronavirus disease (SARSCoV-2) appeared in 2019 was confirmed as pandemic by the WHO on 11 March 2020. Stay-at-home order had an impact on consumers’ food purchase habits, as people around the world were able to leave their homes solely in extremely severe or urgent cases. In our research, we delve into the impact of COVID-19 pandemic on consumers’ food purchase habits. The research involved 3000 consumers during the first wave of coronavirus. The sample represents the Hungarian population by gender and age. To achieve the research goals, we applied multivariate statistical tools. The findings suggest that the pandemic could not change consumer attitude significantly, but the order of factors influencing purchases changed. Consumer motivation factors were organized into four well-distinguished factors: Healthy, domestic, and environmentally friendly choice; Usual taste and quality; Reasonable price; Shelf life. Due to the lack of outstanding data during segmentation, we developed four segments by hierarchical cluster analysis: Health- and environment-conscious women; Price sensitive young people; Taste-oriented men; Quality-oriented intellectuals. The results confirm that food manufacturers and traders need to be prepared for further restrictions in the future.

## 1. Introduction

Over the past two and a half decades, the number of research related to food purchase has multiplied. One of the important conclusions of these behavioral surveys is that consumers strive to choose the right quality of food during purchase. However, food cannot have the main function of alleviating hunger as a sole function but must also provide people with the nutrients they need to prevent the development of nutrition-related diseases. In addition, food should guarantee the physical improvement and mental well-being of consumers [1,2]. According to Grunert and Wills (2007), persuading consumers to eat healthier is not an obvious responsibility. Their research highlighted that each person appreciates health, therefore they need to be informed about the link between diet and a healthy lifestyle [3]. Research shows that health awareness motivates consumers highly when opting for food, but the quality and taste of food are also key factors [4,5,6,7,8,9,10,11].

Consumers increasingly believe that food they consume contributes to their health directly [12,13,14,15].

### 1.1. Theoretical Background of Food Choice

One of the main research focuses on food choice decisions is the study of consumer behavior types related to obesity [16,17,18,19] in which the research models examine food consumers’ choices. Researchers refer to two basic quantitative models when examining this decision-making process [20,21,22,23,24].

One is the Food Choice Questionnaire (FCQ) in which Steptoe et al. (1995) examined food choice habits through 36 statements [25]. The authors identified nine factors that cover the motivations of those involved in the questionnaire in their food choices. These include health; mood; comfort; a sensory appeal; natural content; price; weight control; awareness and ethical considerations. Milošević et al. (2012) identified eight factors by factor analysis; compared to the original model, health and natural content emerged as one factor [26]. Januszewska et al. (2011) tested the questionnaire in the populations of four countries (Belgium, Hungary, Romania, Filipinos) [27].

Szakály et al. (2018) concluded, based on research conducted on a Hungarian sample, that for Hungarian consumers, sensory properties, price, and convenience factors (convenience of shopping and preparation) are the most important factors when choosing foods [28]. It is an interesting result that brand awareness, i.e., the routine, has a much greater influencing effect on the choices of Hungarian consumers than in other cultures. It is backed by the survey of Markovina et al. (2015) of nine European countries, which reveals that awareness is one of the least important factors in other European cultures [29]. At the same time, health aspects are less considered by Hungarian consumers compared, for example, to Serbian [30], Finnish [31], Belgian, Romanian, and Filipino [27] consumers. Of course, there are gender differences, as the health status of food is relatively more important for women, while awareness has a greater impact on men, as confirmed by the international research mentioned above. Renner et al. (2012) stated in their research those important motivational elements were not covered by the original FCQ study [32]. Using 78 statements in their TEMS model (The Eating Motivation Survey), they developed 15 factors (1. pleasure, 2. habits, 3. need and hunger, 4. health, 5. comfort, 6. indulgence, 7. traditional eating, 8. natural content, 9. social life, 10. price, 11. visual temptation, 12. weight control, 13. emotion regulation, 14. social norms, 15. social image), which cover, among other things, social and psychological aspects. Renner et al. (2012) based on their research on a German sample showed that participants identified motivation factors as the Liking, the Habits, the Need and the Hunger, and Health as the most common factors influencing their eating behavior [32]. In contrast, less important factors influencing choice are Social Image, Social Norms, and Affect Regulation. The results are consistent with the findings by Steptoe et al. (1995) based on FCQ [25].

Among the various models, Three Factor Eating Questionnaire (TFEQ) model as the most commonly used research tool has been developed to evaluate eating behaviors of different populations [33,34,35]. Originally, the survey with 51 statements was a self-assessment questionnaire whose main purpose was to analyze eating and related decision-making and behavioral processes among overweight individuals. TFEQ has been adapted by several researchers from several countries [36,37,38]. There are also shorter versions of the questionnaire that consist of fewer statements. The most common of these are the 21-item (TFEQ-R21) and 18-item (TFEQ-R18) versions, which are the most popular in psychology and sociology [39]. Karlsson et al. (2000) developed an 18-factor version of the original three-factor dietary questionnaire (TFEQ) (TFEQ-R18) [40]. TFEQ-R18, similar to TFEQ and TFEQ-R21 versions, is based on three factors: cognitive restraint (CR), emotional eating (EE), and uncontrolled eating (UE). The shortened Three-Factor Eating Questionnaire (TFEQ-R18) is one of the most widely used tools for evaluating eating behavior. Most TFEQ-R18 studies aim to examine overweight and obese individuals [41,42,43,44], while FLVS Study Group researchers were given the same factor structure when examining individuals with normal body weight as in the previous research by Hyland et al. (1989) [45], thus finding that the 18-statement model is also valid when examining non-obese individuals. Among their results, differences between gender and youth and adults were highlighted [46].

### 1.2. Food Purchase Habits during the Pandemic

On 11 March 2020, the WHO confirmed coronavirus disease (SARSCoV-2), which appeared in 2019, to be a pandemic [47]. The emergence and rapid spread of the COVID-19 pandemic poses a significant challenge to health systems around the world [48]. In its global recommendation (2020), the WHO proposed that national governments impose restrictions to curb the spread of the infection. Despite the measures, the number of confirmed cases was already above 3.5 million on 5 May 2020, and the number of confirmed deaths exceeded 250,000 [49]. Stay-at-home orders have affected not only social relationships but also the functioning of businesses and corporations, as well as the physical activity and eating-buying habits of the population, as people worldwide could only leave their homes in extremely severe or urgent cases [50].

A pandemic can fundamentally change food purchase habits. In EY-Future-Consumer-Index (2020) study, it is claimed that four major trends in consumer behavior emerged during COVID-19 [51]. These are as follows: 1. (CUT DEEP) ’Spend less.’: This segment is made up particularly of people over the age of 45 whose employment situation has been most affected by the pandemic. 2. (STAY CALM, CARRY ON) ‘Stay calm and don’t change.’: These consumers were not affected by the effects of the pandemic; therefore, they did not change their shopping habits. 3. (SAVE AND STOCKPILE) ‘Save and store.’: Consumers in this group are significantly concerned about the health of their families and the long-term development of their financial situation. 4. (HIBERNATE AND SPEND) ‘Hibernate and Spend More!’: The group consists mainly of 18–44-year-old people who have a duality in terms of consumer habits. On the one hand, they are concerned about the pandemic, but only 40% said they purchase less often due to this fact. However, their shopping habits have changed significantly, with 46% stating that brands are more important during shopping than before. The study examined the consumer habits of 4859 individuals in the United States, Canada, the United Kingdom, France, and Germany. Overall, 42% of the respondents believed that the way they purchased had fundamentally changed as a result of COVID-19. The results of Güney and Sangün (2021) suggested that the changes were mainly related to fear of price increases, stockpiling, the purchase of usual and excessive quantities of food, food availability and a sense of security, as well as conscious food waste and natural foods and products related to packaging [52].

As a result of the closures, several studies found that individuals’ food choice and consumption habits have changed. According to the findings of Marinković and Lazarević (2021), the fear of the negative effects of the virus and the precautions set up for prevention influenced food purchase habits of consumers significantly [53]. Marty et al. (2021) studied how changes in food choice motivations are related to changes in food quality during restrictions compared to the pre-pandemic period [54]. Nine food motivations were examined: 1. health, 2. comfort, 3. sensory attractiveness, 4. natural content, 5. ethical concern, 6. weight control, 7. mood, 8. awareness, 9. price. Shen et al. (2020) examined the same nine motivations whether emotional eating is affected by high (73.6%) stress levels among respondents as a result of COVID-19 [55]. The Dutch Eating Behavior Questionnaire (DBEQ) [56] and the Food Choice Questionnaire (FCQ) were applied in the evaluation. Rodríguez-Pérez et al. (2020) examined changes in the eating habits among the Spanish adult population during the closures [22]. In general, out of the motivations, mood, health, comfort, and natural content were the common motivational factors. The growing popularity of digital trade has been examined in a study published by McKinsey & Company that analyzed consumer behavior in 45 countries [57]. According to the publication, online customer base growth is on average 30%. Due to increased uncertainty, consumers spend most of their expenditure on basic products and reduce discretionary spending (on non-basic products and services).

Szonda Ipsos Media, Opinion and Market Research Institute (https://www.ipsos.com/hu-hu/elerheto-valsag-hatasait-fogyasztoi-szempontbol-vizsgalo-kutatassorozat-elso-heti-riportja, accessed on 8 June 2021) has been measuring the effects of coronavirus epidemic on consumer behavior in Hungary from April 2020 on a weekly basis, i.e., from the first days of the stay-at-home order. The situation emerged as a result of the coronavirus reshapes the lifestyle, habits, behavior, and decision-making mechanisms of consumers fundamentally. As a result of the pandemic, half of the adult domestic population (52%) do not leave their homes at all, while only one in twelve still leaves their homes as usual (8%). In March–April 2020, Soós conducted an online questionnaire survey on changes in domestic consumer behavior as a result of COVID-19 [58]. In his study, she explains that consumers choose smaller stores and markets instead of larger malls, and the role of online shopping has increased. The extent of the decrease in personal purchases is well-shown by the database of the Hungarian Central Statistical Office. Based on data of the Hungarian Central Statistical Office (2021), we can see that while in March 2019 the turnover of domestic retail stores increased by 4.9%, in April by 8.5% and in May by 5.0% compared to the same period of 2018, by 2020 after an increase of 3.5% in March, the turnover of retail stores decreased by 10.2% in April and by 2.1% in May compared to 2019 [59].

The COVID epidemic has created a new situation for both food supply companies and consumers on the demand side, patterns of which have not been available so far. Thus, as a research problem, we attempted to assess what patterns of food purchasing behavior can be observed among consumers in the event of a hitherto unknown emergency. That is, how an external constraint changes food-purchasing behavior.

A review of the literature has revealed that so far, few studies have examined the changes in consumers’ motivations for buying food in the context of the COVID epidemic. Consequently, our most crucial goal is to examine the motivations of the Hungarian population for food purchases during the restrictions caused by the first wave of COVID-19. In our study, we analyze whether the motivations of the Hungarian population to buy food have changed as a result of the emergency generated by the pandemic. For comparability, we assessed the pre-pandemic situation, which we compared with the situation in the first wave. Ideally, our findings would provide guidance to food producers on how to change their products, sales channels, and communications in response to the expected additional needs, in line with the changing needs of consumers.

## 2. Materials and Methods

### 2.1. Sampling

Experts from the National Food Chain Safety Office, the Institute of Marketing and Trade of the Faculty of Economics of the University of Debrecen, and the TÉT Platform conducted a survey examining the impact of the epidemiological situation on food consumption in the form of an online questionnaire during COVID-19. 

The survey took place from 2 to 19 May 2020. A link generated from a questionnaire editing interface was shared on the most popular social network in Hungary in the form of a post. Paid ad campaigns were associated with that post. These campaigns were advertised to all target groups with the same image and wording. In order to create a representative sample in terms of gender and age, five parallel advertisements by age groups were placed in the first wave. The duration of the first wave of ads was four days, from Friday morning to the following Tuesday morning. Based on the data of the incoming questionnaires, the socio-demographic distribution of the respondents was continuously monitored. Then, in the next wave of advertising, the demographic groups, up to that point representing a smaller proportion of respondents were targeted. This second wave was a more concentrated campaign in terms of time, with a maximum of two days. The purified sample consists of 3000 items. The sample was weighted so that the sample could represent the Hungarian adult population aged 18 and older by gender and age. As the number of the examined age group in Hungary is approximately 8000 thousand [59] and with a 95% confidence level and a 5% margin of error, based on the work of Gill and Johnson (2002), the required sample size is 385, the sample size is appropriate for the study objectives [60].

### 2.2. Structure of the Questionnaire

The quarantine questionnaire consisted of three major blocks: 1. food purchase, 2. nutrition, food consumption and physical activity, 3. food supply and safety. The block examining food purchasing habits was further divided into three sub-blocks: 1.1. purchase motivations, 1.2. frequency of shop visits, 1.3. purchase volume of certain food categories. 

The present article discusses only the results concerning food purchasing motivations. The set of questions on motivations was based on a model used by the authors in a previous study examining food choice motivations. [61]. This part of the questionnaire consisted of three parts. In the first part, respondents had to answer how important various factors influencing purchasing were in the pre-epidemic period. The second part of questions analyzed the same factors influencing purchasing, only in the first wave of the pandemic. Factors influencing the purchase used are illustrated in Table 1.

In the last part of the block, we questioned about the socio-demographic characteristics of the respondents (gender, age, type of settlement, region, highest level of education completed, subjective sense of income, perceived health, and environmental awareness). Table 2. shows the percentage distribution of the socio-demographic groups of the individuals involved in the survey and the population composition according to the previously mentioned two factors.

### 2.3. Data Analysis

Data analysis was performed with SPSS mathematical-statistical analysis software. In order to achieve the research goals, we used both descriptive and multivariate statistical tools. Among the descriptive methods, mean, standard deviation, coefficient of variation, and skewness were calculated. We carried out exploratory factor analysis at first out of the multivariate statistical methods. The purpose of Empirical Distribution Function Statistics (EDF) was to explore what factors can influence purchasing factors during a pandemic. Afterwards, we examined the reliability of the scales used within the measurement model of the revealed latent variables, using Cronbach’s alpha and composite reliability indicators. Segmentation was performed by cluster analysis, which consisted of two main steps: in the first step we determined the number of clusters/segments by hierarchical cluster analysis, then we performed the cluster analysis using K-means method in such a way that the cluster mean was left to the applied program. Cross-tabulation analysis and simple hypothesis tests were used to examine the clusters.

## 3. Results

### 3.1. Examining Customer Behavior before and during the First Wave 

We first asked how important the aspects listed below were for respondents when purchasing food before coronavirus. The results are shown in Table 3.

The results showed that two factors reached value above 4, these included taste and consistent quality. In this case, the standard deviation and coefficient of variation are particularly low, and the skewness, especially in case of taste, is strongly negative. This means that respondents consider these aspects to be more important. This is followed by the popular and usual brand, favorable price, integration into a healthy diet and the shelf life of the food. The search for Hungarian products and local products/food from small farm is a relatively important aspect, but environmentally friendly packaging is also of medium importance. Bio/organic origin and advertising are still at the bottom of the list.

In the following, we examined how the direction of consumers’ thinking about food purchases changed during coronavirus. Related data are shown in Table 4.

It can be clearly seen that the first two ranks did not change compared to the pre-pandemic situation. Taste and consistent quality are basic expectations of consumers, and a crisis or epidemic cannot change that. Shelf life of the food, on the other hand, has advanced three places, the mean increased, the standard deviation and coefficient of variation decreased, and consumers agree with this influencing factor to a greater extent than in the pre-pandemic period. At the same time, the popular and usual brand declined, indicating that the brand and its usual aspect are not so important to consumers during a crisis, it is easier for consumers to give them up, often out of compulsion. Similarly, the packaged product moved up the hierarchy, which typically moves with shelf life. The Hungarian origin of the product maintained its position during the pandemic, but it could not improve on the importance mean. Environmentally friendly packaging, trademark, organic origin, and advertisement all ranked in the last places.

### 3.2. Factor Analysis

Based on the exploratory factor analysis, we were able to identify four factors (Table 5). During the analysis, we obtained a model with a high explanatory power of 65.093%. The first, strongest factor was named ‘Healthy, Domestic, and Environmentally Friendly Choices,’ which explains 27.242% of the variance. High factor weights suggest that the value dimension shapes shopping habits of Hungarian consumers to a large extent and sharply separated from the others. Regarding the Skewness indicator, it can be concluded that the distribution is significantly skewed to the left (Skewness = −0.187), i.e., Hungarian consumers consider this way of thinking to be more relevant for themselves. The second factor is ‘Usual taste and quality’, which explains 9.919% of the variance, related to the taste of the products and their constant quality. The factor is skewed to the left (Skewness = −0.864), i.e., Hungarian consumers are more typical of these statements. The third factor is ‘Favorable Price’, which includes claims about product price developments. The factor explains 9.749% of the variance, and the high factor weights suggest that the dimension shapes the thinking of Hungarian consumers to a large extent. The left skewness of the factor is also expressed (Skewness = −0.439). The fourth and weakest factor is ‘Shelf life’ which explains 7.724% of the variance. With respect to the skewness of the factor, it can be concluded that the distribution is skewed to the left (Skewness = −0.200), i.e., this way of thinking is also considered more relevant for themselves in the study. Convincing advertisement as a factor influencing purchasing was based on several factors and appeared with a low factor weight, therefore we deleted it from the factors.

### 3.3. Examination of the Suitability of the Measurement Tool

Before segmentation, we had to test the suitability of the measurement tool for further surveys. Reliability was examined with the Cronbach’s alpha index and the composite reliability (CR) index, based on which our measurement tool can be considered reliable, and reliability cannot be further increased by removing items [64,65]. The results of the reliability test are summarized in Table 6.

### 3.4. Segmentation 

During segmentation, the segmentation criteria were the previously defined attitudes (factors): Healthy, domestic, and environmentally friendly choice, Usual taste and quality, Favorable price, Shelf life. Once our data proved suitable for segmentation, we determined the number of clusters by hierarchical cluster analysis and examined whether we had outliers. Since no outliers were found and the number of segments was determined in 4 clusters, we ran the cluster analysis using K-means method, during which the determination of the cluster means was left to the algorithm. The formed clusters differ significantly from each other (*p* < 0.01) based on the analysis of variance, i.e., the result of the segmentation is valid. We were able to involve a total of 2748 people in the clusters. In the following, a detailed characterization of each cluster is performed in accordance with the objectives of the research.

- Cluster 1–Health and environment-conscious women

The proportion of the group is 30.8% (847 people) among all respondents. Within the segment, women are significantly over-represented (37.4%) and dominated by the oldest (over 60–40.5%). Within the group, the number of 18–29-year-old and 30–39-year-old people (23.9% and 21.6%, respectively) is low. The group has a higher proportion of those with vocational education/technical education (35.7%) and high school graduates (34.5%). More than 60% of the cluster is made up of people living in better financial conditions, who, in addition to their daily livelihood, can save to a greater or lesser extent. Those whose income decreased significantly as a result of the pandemic (36.1%) are in the majority, while those who ’increased compared to the past’ (16.2%) represent a smaller number in the cluster. They typically live in cities (34.1%) and villages (32.5%). The members of the cluster are characterized by health and environmental awareness; ’Very health conscious’ (35.4%) and ’Very environmentally conscious’ (33.5%) dominate.

This cluster is characterized by multilevel awareness, as they rated most of the motivational factors as the highest. It is also their characteristic that they monitor the shelf life, packaging, and price of the food. In addition, they typically prefer products that are already popular or usual, but also consider flavors associated with a particular product and the consistently high quality. They also make sure that these products can be easily integrated into a healthy diet. The cluster significantly overestimates the Hungarian origin and the local products/food from small farms compared to other segments. This is backed by the predominance of health- and environment-conscious individuals in the cluster.

- Cluster 2–Price sensitive young people

The proportion of the group is 16.0%, i.e., 439 people, making it the smallest cluster. The proportion of women (15.8%) and men (16.1%) in the segment is almost the same. The cluster is dominated by those aged 18–29 (22.0%). In the group, people over the age of 60 (11.9%) and those aged 40–59 (15.3%) are present in low numbers. The segment is dominated by those with a ‘grade 8 education’ (33.3%), while the number of people with a tertiary education is low (13.0%). Examining their income situation, they are characterized by rather a worse financial situation. As a result of the pandemic, those who lost their previous income (39.5%) are represented in the group in an outstanding proportion. Typically, urban residents make up the cluster (49.66%). The group is not typically health and environmental conscious. ‘Not health-conscious at all’ (38.7%) and ‘Not environmentally conscious at all’ (42.1%) dominate.

For those in the cluster, the price of the products is extremely important, and advertising related to the product is not important at all. The shelf life and packaging of food is also important. They can be considered less health and environmental conscious and the high-quality food is less important, as well as the bio/ecological origin, and this group is the least motivated by the Hungarian origin and the purchase from local producers.

- Cluster 3–Taste-oriented men

Based on its size, it is the second largest cluster (27.1%, 744 people) of the four. Gender is strongly dominated by men (32.8%) while the rate of women is lower (21.9%). Among the age groups, those aged 18–29 (34.9%) and 30–39 (33.8%) stands out slightly. People over the age of 60 are present in lower numbers in the group (20.4%). The segment is dominated by a maximum of ‘8 grade education’ (36.4%). Other education groups make up the cluster in almost equal proportions. Within the group, those in the average financial situation (they can make ends meet but can save little—31.2%) and (Just enough to make ends meet, but unable to save—26.6%) play a decisive role. Within the segment, the majority are those whose income increased during the pandemic (35.1%). According to the place of residence, the population of the capital (28.8%), the inhabitants of the village (27.2%) and the city (25.9%) occur in almost the same proportion within the group. In the group, ‘Mostly not health-conscious’ (36.1%) and ‘Mostly not environmentally conscious’ (45.0%) people stand out in the group.

For those in the cluster, convincing advertisements are not important at all, but the taste and enjoyment value associated with the products is important. It is not important, either, that the product they buy is packaged, and it is less characteristic to pay attention to the environmentally friendly characteristics of the product they buy. The health factor of the product as well as its organic/ecological origin are not considered when making a purchase decision.

- Cluster 4–Quality-oriented intellectuals

According to the subjective sense of income, those with slightly better income than average (they make a living from it, but they can save little—36.3%), and those with much better income than average (they make a good living from it and can save—32.7%) dominate in the group. The group was over-represented by those whose income increased during the pandemic compared to the previous period (35.1%). Within the group, the residents of the capital (28.5%), those living in the village (26.1%) and the city (24.5%) are almost equally represented. ‘Mostly not health-conscious’ (27.9%) and ‘Very health-conscious’ (26.1%) appear in the cluster in almost the same proportion. This duality can also be observed when examining subjective environmental awareness.

Convincing advertisement related to the product, or favorable or discount price are less important, which may be related to better financial situation. Typically, it is the flavor associated with the product and consistent quality that influence them when shopping. To a lesser extent, but the packaged and durable nature of the product they want to buy is also important; however, environmentally friendly packaging is moderately important. Slight brand loyalty characterizes the segment, while integration into a healthy diet is somewhat more important than moderate.

## 4. Discussion

In our study, we undertook to present the influencing effect of the restrictions experienced during the first wave of COVID-19 on food choice and food purchase habits of Hungarian consumers. We found that the pandemic could not change consumer thinking; however, the order of some factors changed. Compared to the period before closure, the shelf life and packaged nature of the products are those that came first in order of priority. This result is consistent with the results of Güney and Sangün (2021) [52], where the authors found that the mode of product packaging influenced food motivation significantly during the pandemic. According to research by European Institute of Innovation & Technology (EIT-Food, 2020), since the outbreak of COVID-19, 33% of consumers have paid more attention to the packaging of the product they intend to choose [66]. However, while in our study packaging was an important factor mainly during the pandemic due to the shelf life of food, in the EIT-Food (2020) research, the need for environmentally conscious packaging also appeared to a minimal extent [66]. In our research, the issue of environmental awareness was somewhat pushed into the background during the pandemic period. The weight of environmental sustainability in food packaging also appears to be declining compared to the pre-pandemic period, according to an International Food Information Council (IFIC, 2021) study, but their research shows that more than half of consumers still consider environmentally friendly packaging important [67]. Of course, packaging is also related to the shelf life of the products. Fear of this can also be observed in the IFIC (2021) Food and Health Survey, with 40% of consumers being worried about the early expiry of the shelf life of the food they buy [67].

Examining customer behavior in the pre-pandemic period, as well as during the first wave of the epidemic, ‘product-related flavors’ aspect was the most important to consumers. This result is consistent with a previous study by Glanz et al. (1998) [18], where the authors suggest that people are more likely to choose foods during purchase, they find delicious. This kind of perception can also be observed in the IFIC (2021) study, as 82% of those surveyed identified taste as the strongest motivation to buy [67]. Although the value of motivation for product taste decreased compared to the previous year, it is still the factor that most influential regarding consumers’ final decision. This was followed by the price of the products (66%), their health property (58%), the convenience of shopping (52%) and the environmental sustainability of the products (31%). In our study, the role of brand loyalty decreased during the pandemic, which is consistent with the study by Arora et al. (2020) [57]. According to their results, when consumers could not find their preferred product s) in their usual and popular store due to the pandemic, they changed their purchasing behavior and other brand(s) or store(s).

According to EIT-Food (2020) research, the persistently favorable prices and availability of products appear to be re-evaluating, and in the post-pandemic period, factors related to the convenience of food purchases and food prices come to the fore [66]. According to our study, the motivational power of the special price and the persistently low price of products decreased overall, but the influencing role of food prices in certain social strata strengthened. This group includes particularly young people who lose their jobs and incomes.

Hungarian origin maintained its position during the pandemic. According to Hobbs (2020), it seems likely that demand for local foods after COVID-19 will increase in the short to medium term, as the importance of local foods is a well-established consumer trend that, according to Cranfield et al. (2012), has economic, social, environmental, and health benefits [68,69].

Table 7 summarizes the results of research examining the food choice motivations of Hungarian consumers based on different models. 

According to Szakály (2008), 86% of Hungarian consumers rarely or never give up on the good taste of food for the sake of its healthiness. Based on the author’s research, it can be stated that European consumers prefer the enjoyment value over the healthiness of the products (taste orientation), as opposed to Asian consumers, who have a preference for nutritional benefits [71]. As can be seen from Table 7, even such an extraordinary epidemic situation could not affect the priority of taste in the Hungarian market. However, it should be noted that in recent years, a favorable attitude towards healthy eating has become more and more pronounced in Hungary, as shown by the motivational rankings of the various models. While at the time of the study carried out based on the FCQ model (2018), health was among the least important aspects, based on the TEMS model (2019) and the present research, it ranks fourth to fifth among motivations. Thus, although Hungarian consumers did not give up on taste during the quarantine, they were trying to create a diet as healthy as possible. 

The fact that storage life and the packaged nature have come to the fore can be attributed to the panic purchase typical of the first wave of the epidemic. In addition, the opportunity to make a good purchase remained a major motivation. It is worth noting that ethnocentric thinking is also gaining ground.

## 5. Conclusions

Results indicate that the motivations of Hungarian consumers to choose food did not change in the first wave of COVID-19; however, the order of preference changed. During the period considered, the excellent taste of the products and the consistently high quality associated with the selected products retained their leading position, ahead of health considerations. This order of priority also determines the directions and opportunities of product development. For Hungarian (and generally European) consumers, existing products with a familiar taste need to be enriched with functional, health-promoting features, thus nudging them towards healthier choices.

As a result of the lockdown, the importance of the popular, familiar brand has decreased, suggesting that consumers are more likely to give up their regular brand and product if they experience a crisis. The shorter distribution channels have become slightly more appreciated, which has proved to be beneficial for food produced in Hungary.

This result was also supported by factor analysis. Customer motivational factors were divided into four well-distinguished factors, which we referred to as: Healthy, domestic, and environmentally friendly choices; Usual taste and quality; Reasonable price; Shelf life, out of which it was the price factor that appeared to be more important during quarantine, suggesting that its influential role was greater in this period. Nearly 20% of the population lost their income, or else their income fell significantly during the period of restrictions, while slightly more than 20% expect their household income to decline in the future. Accordingly, it can be assumed that the influencing factors related to food prices will come to the fore in the future. 

Another important outcome of our survey is that consumer segments have become basically divided into three parts; along with the demanding and ethnocentric shoppers, those favoring taste and cosmopolitans, as well as rational shoppers, were present. It is worth highlighting the demanding, quality-oriented customers, as they were the ones who lived extremely consciously during the quarantine period: they paid attention to the use of food, studied food labels more thoroughly and tried to eat even healthier than before, and they can also be characterized by a high level of patriotism. This multi-level awareness makes them fully suited to act as a kind of reference group for those who want to follow a healthy and conscious lifestyle, as well as to be a primary target group for companies. Of course, taking priorities into account, additional target groups can be identified, such as those who are enjoyment value-oriented or price-sensitive, which allows for even more specific strategies.

The positive result of our research is that the motivational factors and clusters identified enable us to predict the reactions of the population, thus helping organize the supply chain and increase the reaction speed of companies in other, similar situations.

The limitation of the study is the lack of study models. For the sake of comprehensive research, it would therefore be necessary to associate a model or models with the study, which would facilitate comparison with research results on a similar topic.

## Figures and Tables

**Table 1:** Factors influencing purchase and their measurement (N = 3000).

Factors Influencing Purchase	Measuring Scale
Constantly high quality	A scale from 1–5, where 1–not important at all; 5–extremely important.
Flavors associated with the product	
Popular and usual brand	
Convincing advertisement	
Favorable price	
Integration into healthy diet	
Discount price	
The food was produced in Hungary	
Bio/ecological origin	
Environmentally-friendly packaging	
The product is packaged	
Shelf life of the food	
Local product/food from small farms	
It has trademark	

Source: [61].

**Table 2:** Distribution of the sample according to the most important background variables (N = 3000) and population composition according to representative variables.

Background Variables	Sample Distribution		Population Distribution ^1^
Female	1587	52.9	52.2
Male	1413	47.1	47.8
18–29	507	16.9	17.2
30–39	483	16.1	16.0
40–59	1044	34.8	34.7
60–	966	32.2	32.1
Budapest	1059	35.3	
Other towns	1530	51.0	
Village	411	13.7	
Central Hungary	1515	50.5	
Southern Great Plain	282	9.4	
Northern Great Plain	267	8.9	
Northern Hungary	261	8.7	
Central Transdanubia	252	8.4	
Western Transdanubia	243	8.1	
Southern Transdanubia	180	6.0	
Primary school	36	1.2	
Vocational school	219	7.3	
High school	984	32.8	
Higher education	1761	58.7	
Can live on it but can save little	1215	40.5	
Can live on it very well and can also save	993	33.1	
Just enough to live on but cannot save	624	20.8	
Sometimes cannot make ends meet	78	2.6	
Have regular financial problems	27	0.9	
Not known/No answer	63	2.1	
Mostly health conscious	1564	52.1	
Health-conscious and not health-conscious	834	27.8	
Very health conscious	296	9.9	
Mostly not health conscious	209	7.0	
Not health conscious at all	67	2.2	
Not known/No answer	30	1.0	
Mostly environmentally conscious	1723	57.4	
Both environmentally conscious and not	621	20.7	
Very environmentally conscious	469	15.6	
Mostly not environmentally conscious	141	4.7	
Not environmentally conscious at all	26	0.9	
Not known/No answer	20	0.7	

Note: ^1^ Source of data [62,63].

**Table 3:** Importance of purchasing factors when buying food before coronavirus (N = 3000).

Purchasing Factors	Statistical Indicator			
	Mean ^1^	Standard Deviation	Relative Standard Deviation, %	Skewness
1. Flavors associated with the product	4.35	0.752	17.29	−1.415
2. Constantly high quality	4.01	0.895	22.32	−0.876
3. Popular and usual brand	3.92	0.936	23.88	−0.768
4. Favorable price	3.85	0.977	25.38	−0.768
5. Integration into a healthy diet	3.83	1.052	27.47	−0.790
6. Shelf life of the food	3.55	1.081	30.45	−0.425
7. Discount price	3.52	1.109	31.51	−0.480
8. The food was produced in Hungary	3.41	1.252	36.72	−0.448
9. Local product/food from small farm	3.05	1.277	41.88	−0.101
10. Family-friendly packaging	3.04	1.220	40.13	−0.115
11. It has trademark	2.90	1.291	44.52	0.003
12. Bio/ecological origin	2.66	1.238	46.54	0.175
13. The product is packaged	2.54	1.246	49.06	0.364
14. Convincing advertisement	1.67	0.832	49.82	1.226

^1^ Results were rated on a scale of 1 to 5, with a value of 1 for ‘Not important at all’ and a value of 5 for ’very important’.

**Table 4:** Importance of purchasing factors when buying food during coronavirus (N = 3000).

Purchasing Factors	Statistical Indicator			
	Mean ^1^	Standard Deviation	Relative Standard Deviation, %	Skewness
Flavors associated with the product	4.14	0.848	20.48	−1.055
2. Constantly high quality	3.98	0.943	23.69	−0.952
3. Shelf life of the food	3.92	1.089	27.78	−0.956
4. Favorable price	3.67	1.095	29.84	−0.568
5. Integration into healthy diet	3.64	1.131	31.07	−0.688
6. Popular and usual brand	3.59	1.080	30.08	−0.549
7. The food was produced in Hungary	3.33	1.337	40.15	−0.398
8. Discount price	3.33	1.233	37.02	−0.321
9. The product is packaged	3.17	1.429	45.08	−0.213
10. Local product/food from small farm	3.05	1.339	43.90	−0.137
11. Family-friendly packaging	2.89	1.288	44.57	0.000
12. It has trademark	2.79	1.342	48.10	0.117
13. Bio/ecological origin	2.61	1.275	48.85	0.246
14. Convincing advertisement	1.56	0.821	52.63	1.555

^1^ Results were rated on a scale of 1 to 5, with a value of 1 for ’Not important at all’ and a value of 5 for ’very important’.

**Table 5:** Results of exploratory factor analysis (N = 3000).

Factors Influencing Purchase	Healthy, Domestic, and Environmentally-Friendly Choice	Usual Taste and Quality	Favorable Price	Shelf Life
Local product/food from small farm	0.817			
The food was produced in Hungary.	0.796			
Bio/ecological origin	0.736			
Environmentally packaging	0.704			
It has trademark	0.685			
Integration into a healthy diet	0.508			
Convincing advertisement				
Flavors associated with the product		0.788		
Constantly high quality		0.704		
Popular, usual brand		0.523		
Favorable price			0.897	
Discount price			0.791	
The product is packaged				0.925
Shelf life of the food				0.437

Extraction method: Maximum Likelihood; Rotation method: Varimax rotation; Rotation converged in 5 iterations; KMO = 0.820 (incredibly good); Bartlett: (Approx. Chi Sq.) 14,477.24; (Sig.) 0.000; Communalities: 0.118–0.884; Cumulative explained variance: 65.093; N = 3000.

**Table 6:** Reliability of the measuring instrument.

Factors	Cronbach’s Alpha Index	Composite Reliability
Healthy, domestic, and environmentally-friendly choice	0.878	0.891
Usual taste and quality	0.716	0.608
Favorable price	0.829	0.444
Shelf life	0.595	0.210

Source: based on own calculation.

**Table 7:** Hungarian consumers’ food choice motivations based on different models in the pre-coronavirus period and during the first wave.

Model (Year of Questioning)	Factors in Order of Importance
FCQ, 2018 (Food Choice Questionnaire) [28]	Sensory Appeal, Price and Purchase Convenience, Preparation Convenience, Familiarity, Health and Natural Content (+Weight Control), Mood, Ethical concerns.
TFEQ, 2019 (Three Factor Eating Questionnaire) [24]	Emotional Eating, Uncontrolled Eating, Cognitive Restraint Factor
TEMS, 2019 (Eating Motivation Survey) [70]	Pleasure, Habits, Need and Hunger, Health, Comfort, Traditional Eating, Indulgence, Natural Content, Price, Social Life, Weight Control, Visual Temptation, Social Image, Social Norms, Emotion Regulation.
Before COVID-19 (2020)	Flavors associated with the product, Constantly high quality, Popular and usual brand, Favorable price, Integration into a healthy diet, Shelf life of the food, Discount price, The food was produced in Hungary Local product/food from small farm, Family-friendly packaging, It has trademark Bio/ecological origin, The product is packaged, Convincing advertisement
During the first wave of COVID-19 (2020)	Flavors associated with the product, Constantly high quality, Shelf life of the food, Favorable price, Integration into healthy diet, Popular and usual brand, The food was produced in Hungary, Discount price, The product is packaged, Local product/food from small farm, Family-friendly packaging, It has trademark, Bio/ecological origin, Convincing advertisement

## Data Availability

The data presented in this study are available on request from the corresponding author. The data are not publicly available due to ethical restrictions.
